# Role of Stro1^+^/CD44^+^ stem cells in myometrial physiology and uterine remodeling during pregnancy^†^

**DOI:** 10.1095/biolreprod.116.143461

**Published:** 2016-12-23

**Authors:** Aymara Mas, Lauren Prusinski, Qiwei Yang, Patricia Diaz-Gimeno, Lelyand Stone, Michael P Diamond, Carlos Simón, Ayman Al-Hendy

**Affiliations:** 1 Department of Obstetrics and Gynecology, Augusta University, Augusta, Georgia, USA; 2 Fundacion Instituto Valenciano de Infertilidad (FIVI), Department of Obstetrics & Gynecology, School of Medicine, Valencia University and Instituto Universitario IVI/INCLIVA, Valencia, Spain; 3 Igenomix, Valencia, Spain; Instituto de Investigación Sanitaria INCLIVA, Valencia, Spain; Department of Pediatrics, Obstetrics, and Gynecology, Universidad de Valencia, Instituto Universitario IVI, Valencia, Spain; Department of Obstetrics and Gynecology, School of Medicine, Stanford University, Stanford, CA

**Keywords:** myometrial stem cells, Stro1/CD44, pregnancy, hypoxia

## Abstract

Regulation of myometrial functions during pregnancy has been considered the result of the integration of endocrine and mechanical signals. Nevertheless, uterine regeneration is poorly understood, and the cellular source within the gravid uterus is largely unexplored.

In this study, we isolated and quantified the myometrial stem cells (MSC) population from pregnant female Eker rat uteri, by using Stro1/CD44 surface markers. We demonstrated that prior parity significantly increased the percentage of Stro1^+^/CD44^+^ MSC because of injured tissue response. Interestingly, we established that Stro1^+^/CD44^+^ MSC respond efficiently to physiological cues when they were treated in vitro under different dose-dependent pregnant rat serum.

Previous studies reveal strong regulatory links between O_2_ availability and stem cell function. Based on these premises, cell proliferation assays showed that isolated Stro1^+^/CD44^+^ MSC possess a higher proliferative rate under hypoxic versus normoxic conditions. We also detected a total of 37 upregulated and 44 downregulated hypoxia-related genes, which were differentially expressed in Stro1^+^/CD44^+^ MSC, providing an alternative approach to infer into complex molecular mechanisms such as energy metabolism, inflammatory response, uterine expansion, and/or remodeling.

Since these cells preferentially grow under low oxygen conditions, we propose that the increase of the rat uterus during pregnancy involves myometrial oxygen consumption, thereby enhancing MSC proliferation. Moreover, pregnancy-induced mechanical stretching results in hypoxic conditions, ultimately creating an environment that promotes stem cell proliferation and further uterine enlargement, which is essential for a successful pregnancy. In summary, all of these data support that rat Stro1^+^/CD44^+^ MSC contribute to uterine enlargement during pregnancy.

## Summary Sentence

The human and murine uterus exhibits a large expansion in size and remodeling over the course of pregnancy. However, at this stage, little is known about the uterine regeneration at cellular level.

Due to limited access to sequentially-timed human pregnant myometrial tissues, we proceeded to address these pivotal questions by using a rat animal model. In this study, we identified and characterized myometrial stem cells (MSC) from pregnant female Eker rat uteri, by using specific surface markers. We also demonstrated their contribution to uterine enlargement during pregnancy under hypoxic conditions. Nevertheless, further studies are needed to elucidate the exact role of myometrial stem cells in healthy pregnancy and also in pathological conditions such as preterm labor.

## Introduction

Human and murine myometrium undergo repeated and dramatic cellular reorganization during pregnancy, as well as postpartum [1]. Seemingly, with the pregnancy-associated abundance in hormonal production, typical myometrial cells can express a new program of genes including hormone receptors, growth factors, and structural and cell-channel proteins [2].

To date, it has been suggested that the regulation of myometrial functions throughout the reproductive lifespan is mainly the result of the integration of endocrine and mechanical signals. Certainly, estrogen (ER) and progesterone (PR) receptors, which are expressed in the myometrium throughout the menstrual cycle [3], play key roles in uterine growth during pregnancy [4,5]. In addition, pregnancy-related genes can reverse to basal levels after parturition [6]. Furthermore, the increase of phagocytosis, lysosomal proteins, and matrix metalloproteinases mediates the involution of the uterus and the resumption of myometrial muscle tone during the postpartum period [7,8].

Nevertheless, beyond these observations, uterine regeneration is poorly understood, and the cellular source that drives such massive expansion and remodeling within the pregnant uterus is largely unexplored. Although several studies have demonstrated that during pregnancy both myometrial hyperplasia (cell number increase) and hypertrophy (cell size increase) contribute to the dramatic enlargement of the uterus in both humans and rodents [1,9,10], it is not clear if involution is a passive process precipitated by the decline in levels of estrogen and progesterone, a decline in their receptors, or whether there is a direct signaling pathway which thereby activates initiation of involution. Moreover, the extent to which human myometrial cell apoptosis or dedifferentiation occurs in postpartum is essentially unknown [2].

Somatic stem cells maintain homeostasis of the tissue by providing a cell reservoir for regeneration and thereby support tissue remodeling and repair [11]. Recently, stem and/or progenitor cells in the human myometrium have been identified and their properties characterized [12,13]. The functions of these myometrial stem cells during pregnancy, however, are not yet understood in great detail. Here, we hypothesize that during pregnancy, the myometrial stem cell population could differentiate into myometrial cells in response to either humoral, environmental, and/or mechanical factors, with a unique hormone-regulated gene profile. Later on, with the onset of parturition, the uterus would start to involute in response to corresponding signaling changes.

A growing body of evidence also supports the concept that low-oxygen conditions permit survival and growth of tissue-specific stem cells in vitro [14] and in vivo [15]. Indeed, human myometrial stem cells preferentially favor a hypoxic environment for proliferation and spontaneous differentiation into smooth muscle cells [12,16], suggesting that this cell population could participate in the enlargement of the uteri during pregnancy under local hypoxic conditions.

Due to limited access to sequentially timed human pregnant myometrial tissues, we proceeded to address these pivotal questions by using a rat animal model. In this study, we isolated and quantified the myometrial stem cell population in pregnant Eker rat [Long Evans; Tsc-2(Ek/+)] uteri by using myometrial stem cell-specific surface markers [12]. We also explored their role in pregnancy-induced remodeling, as well as evaluated the impact of hypoxia on the proliferation and function of these myometrial stem cells.

## Materials and methods

### Animals

Female Eker rats [Long Evans; Tsc-2(Ek/+)] were obtained from an in-house Eker rat colony started by an in-kind gift from Eker rat breeders, Dr. Cheryl Walker, MD Anderson Cancer Center in Smithville, TX. All experiments using these animals were conducted in accordance with guidelines and provisions issued by the National Institutes of Health.

Eker rats carrying a germline mutation in the tuberous sclerosis 2 (*Tsc2*) tumor suppressor gene spontaneously develop uterine leiomyomas with a frequency of 65% between ages 12 and 16 months [17]. Recently, we used this model as part of our ongoing investigations regarding the role of myometrial stem cells in the pathogenesis of these benign tumors [18,19]. However, during the first year of life, these animals definitively demonstrate normal uterine anatomy and physiology, and thus would be an appropriate model for the study of pregnancy-related homeostasis at that age [20].

**Figure 1.**
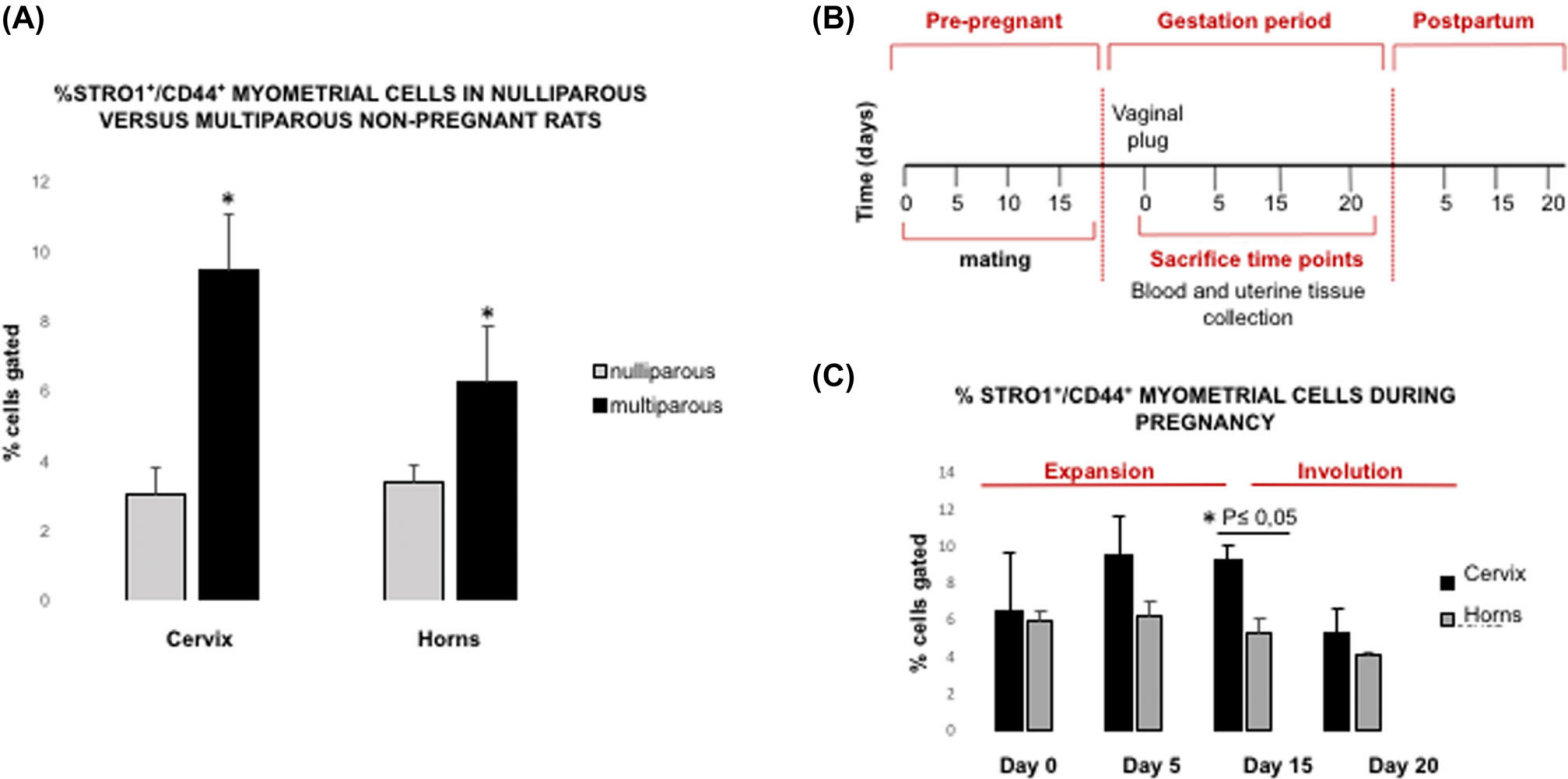
Parity responsiveness in Stro1^+^/ CD44^+^ myometrial stem cell population. (**A**) FACS analysis demonstrating the percentage of Stro1^+^/CD44^+^ myometrial stem cells in nulliparous versus multiparous nonpregnant rats. Unpaired *t* test was used for statistical analysis ^*^P < 0.05. Values are expressed as mean ± SEM for four independent animals per condition. (**B)** Timeline of the experimental methods: female rats at 3 months of age were naturally mated with fertile males. Pregnant female rats were identified by vaginal plugs. Tissue was collected during different time points of pregnancy (D0, D5, D15, D20) for several purposes. **(C)** Quantification of Stro1^+^/CD44^+^ myometrial stem cells from Eker [Long Evans; Tsc-2(Ek/+)] pregnant rats at days 0, 5, 15, 20 of pregnancy (n = 16). Two-tailed Student *t* test was used for statistical analysis ^*^P < 0.05.

Protocols involving the use of these animals were approved by the Committee on the Ethics of Animal Experiments, Augusta University (Augusta, GA). Twenty-four myometrium samples were collected from 3- to 6-month-old nulliparous (never pregnant) and multiparous (at least one documented prior term pregnancy/parturition) female Eker rats for different cellular and molecular approaches. The reproductive stage of each animal (proestrus, estrus, metestrus, diestrus) was determined by histological examination of the vagina and endometrium as described previously [18,19]. Pregnancy-related time points (gestational days 0, 5, 15, and 20) were determined by observation of a vaginal plug during daily checks of the breeding animals, which indicated day 0 of pregnancy. Animals were weighed every day to track growth as further confirmation of pregnancy following observation of the vaginal plug. The experimental setup is outlined in Figure 1B.

### Timed-pregnant rat serum acquisition

Rat serum from 3-month-old timed-pregnant female Long Evans rats at days 5, 15, and 20 of pregnancy was purchased from Envigo laboratories (Envigo Bioproducts, Indianapolis, IN) and prepared as per their certified standard. In addition, serum from 3-month-old nonpregnant rat was included as a negative control.

### Preparation of primary myometrial cells from Eker rat

Uterine tissues from Eker rats were collected from estrus-matched female nulliparous and multiparous timed pregnant between 3 and 6 months old (n = 8). The myometrial layer was isolated by removing the endometrium and serosa with a sterile scalpel. Myometrial tissues were then washed in calcium- and magnesium-containing Hanks balanced salt solution, HBSS (Life Technologies, Grand Island, NY) with 1% antibiotic-antimycotic solution (Life Technologies, Grand Island, NY), and manually minced into small pieces (∼1 mm^3^). Minced portions were then digested overnight at 37°C by enzymatic means according to our previously published protocol [12] in order to obtain single cell suspension.

### Cell sorting and flow cytometric analysis

Freshly isolated rat myometrial cells were resuspended at a concentration of 1×10^6^ cells/mL in 5% bovine serum albumin (BSA; Sigma-Aldrich, St. Louis, MO) for 30 min to prevent nonspecific staining of cells. First, single cell suspensions were subjected to immunophenotypic analysis in order to exclude the hematopoietic stem cells by using 15 μl of CD45 (eBioscience, San Diego, CA), as well as the endothelial cells by using 10 μl of CD31 (BD Bioscience, San Jose, CA). Similarly, isotype controls were prepared for each antibody in separate aliquots. Subsequently, myometrial cell suspensions were then incubated with the conjugated antibodies, Stro1 (eBioscience) and CD44 (BD Biosciences); diluted in isolation buffer containing phosphate-buffered saline (PBS, Sigma-Aldrich); and supplemented with 0.1% BSA (Sigma-Aldrich) and 2 mM of ethylene diamine tetraacetic acid (Supplemental Table S1). Following the incubation period, cells were transferred into tubes for analysis by fluorescence-activated cell sorting (FACS), using FACSCalibur flow cytometer (BD Biosciences), to determine the percentages of Stro1^+^/CD44^+^ cells (considered the putative myometrial stem cells) and Stro1^−^/CD44^−^ cells (considered primary myometrial cells), through CellQuest Pro (BD Biosciences) and FlowJo software (Tree Star Ashland, OR). The FACSCalibur flow cytometer was equipped with an argon laser (488 nm excitation) and a red diode laser (635 nm excitation). The establishment of gates was based on the staining profiles of the negative control isotypes and unstained cells.

### Cell proliferation assay of Stro1^+^/CD44^+^ myometrial stem cells and Stro1^−^/ CD44^−^ myometrial primary cells under different oxygen conditions

To evaluate the effect of oxygen on cell proliferation, isolated Stro1^+^/CD44^+^ myometrial stem cells were immediately placed on ice in order to maintain the original conditions and the low oxygen conditions before they were seeded for cell culture purposes. In addition, Stro1^−^/CD44^−^ myometrial primary cells were seeded in sixplicate at a density of 937 cells/cm^2^ and grown in Clonetics Smooth Muscle Growth Medium-2 (SmGM-2, Lonza, Walkersville, MD) containing 10% fetal bovine serum (Stem Cell Technologies, Canada) and 1% antibiotic-antimycotic (Life Technologies) in either a hypoxic (2% O_2_, 37°C, 5% CO_2_, 90% humidity) or normoxic (20% O_2_, 37°C, 5% CO_2_, 90% humidity) environment. Twenty-four hours after seeding, respective media were removed from the wells, and 3-(4, 5-dimethylthiazol-2-yl)-2, 5-diphenyltetrazolium bromide (MTT reagent, 5 mg/mL in PBS, Sigma-Aldrich) was added and incubated for an additional 4 h at 37°C. After incubation, MTT reagent was blocked with 99.9% dimethyl sulfoxide (Sigma-Aldrich) and incubated for an additional 15 min. The intensity of the resulting purple color per well was proportional to the number of viable cells. Optical density (OD) was measured at 570 nm with a reference filter of 620 nm using a Synergy HT Multi-Mode microplate reader (BioTek Instruments, Inc., Winooski, VT). Cell proliferation was calculated by subtracting the OD of the control wells from the OD of cell-plated wells, and data were represented as the mean of sixplicate wells ± SEM.

### Cell culture of Stro1^+^/CD44^+^ myometrial stem cells under dose-dependent pregnant rat serum treatment

In order to mimic and assess the endocrine/ paracrine effect on uterine cell growth during pregnancy, dose-dependent cell proliferation assays were performed using different dilutions of pregnant rat serum treatments. For this purpose, sorted Stro1^+^/CD44^+^ myometrial stem cells and Stro1^−^/CD44^−^ myometrial primary cells were seeded in sixplicate at a density of 937 cells/cm^2^ and grown in Clonetics Smooth Muscle Growth Medium-2 (SmGM-2), and supplemented with 5% (full concentration) or 2.5% (half concentration) of serum from nonpregnant and timed-pregnant animals at days 5, 15, and 20, under hypoxic conditions (2% O_2_, 37°C, 5% CO_2_, 90% humidity). Cell proliferation was assessed by 3-(4, 5-dimethylthiazol-2-yl)-2, 5-diphenyltetrazolium bromide (MTT) assay, as per the previously described protocol (Wallert and Provost Lab, MSU Moorhead, Moorhead, MN). Data were represented as the mean of sixplicate wells ± SEM.

### Hypoxia signaling PrimePCR array

Total RNA from hypoxia versus normoxia-grown Stro1^+^/CD44^+^ myometrial stem cells was isolated by using TRIzol Reagent (Invitrogen, Carlsbad, CA), and reverse transcribed into the first-strand cDNA using SuperScript III First-Strand Synthesis System for RT-PCR (Invitrogen). The first-strand cDNA synthesis reaction was primed using random hexamers (Invitrogen). Eleven nanograms of cDNA from each condition: hypoxia versus normoxia-grown Stro1^+^/CD44^+^ myometrial stem cells were loaded per gene into separate Hypoxia Signaling Pathway R96 plates (BioRad Inc., Hercules, CA). Expression levels for a total of 81 genes involved in the hypoxia signaling pathway were detected at the same time with SsoAdvanced Universal SYBR Green Supermix on Bio-Rad CFX96 real-time PCR system. The following thermocycling conditions were used: 1 cycle of 95°C for 10 min, 40 cycles of 95°C for 30 s, and 60°C for 1 min. For data analysis, the comparative method (ΔΔCt) was used to calculate relative quantities of nucleic acid sequence through Bio-Rad CFX manager software.

### Gene validation by real-time PCR

To verify the results obtained by PrimePCR array, a real-time PCR (Bio-Rad CFX96) was performed for three selected upregulated genes and five selected downregulated genes. The mRNA levels of insulin-like growth factor binding protein 3 (*Igfbp3*), matrix metallopeptidase 9 (*Mmp9*), plasminogen activator urokinase (*Plau*), protein BTG1 *(Btg1)*, endothelin 1 *(Edn1)*, eukaryotic translation initiation factor 4E binding protein 1 *(Eif4ebp1)*, early growth response protein 1 *(Egr1)*, and cellular tumor antigen p53 *(Tp53)* were quantified using gene-specific primers (Supplemental Table S2). The mRNA values for each gene were normalized to an internal control 18S mRNA, and the data were analyzed by using Bio-Rad CFX manager software. All experiments were performed in triplicates.

### Statistical and functional analysis

Flow cytometric data were analyzed using FlowJo 8.7.3 software. Statistical computing and graphics were performed using R software 3.1 version [21]. An unpaired Student *t* test with 95% confidence intervals was used for comparative parametric analysis, with a significance level of *P*-value ≤ 0.05 considered statistically significant. In addition, the Mann–Whitney *U* test or/and the Kruskal–Wallis rank-sum test were used as nonparametric analysis to compare two groups or more than two groups, respectively.

Functional annotation and relevance of the genes analyzed for hypoxia signaling PrimePCR array were evaluated using the KEGG mapper tool from the KEGG pathways database [22] by using rattus novergicus (rno) as the model organism. For pathway relationship modeling and visualization, Cytoscape network software 3.3 version was implemented [23].

## Results

### Identification and quantification of the Stro1^+^/CD44^+^ myometrial stem cell population in nulliparous versus multiparous nonpregnant rats

The dramatic uterine enlargement and remodeling process during pregnancy suggests the existence of myometrial stem cells. To identify this cell population in the murine myometrium, we used the Eker rat model, where tissue access and environmental exposures could be well controlled. First, myometrial tissue was separated from endometrium and serosa, minced and enzymatically digested to obtain single cell suspensions, with the absence of hematopoietic and endothelial cells confirmed by using specific cell surface markers CD45 and CD31, respectively (Supplemental Figure S1). Subsequently, myometrial single cell suspensions from nulliparous and multiparous nonpregnant rats were subjected to the FACS analysis using Stro1/CD44 antibody-based sorting. For the purposes of this study, we decided to study the uterine horn region (where the pregnancy is harbored and maintained) separate from the uterocervical region which typically functions to maintain the pregnancy and prevent premature expulsion of fetuses. Our results demonstrated that prior parity significantly increased the percentage of Stro1^+^/CD44^+^ myometrial stem cells in both the uterocervix (9.47 ± 2.18 vs 3.02 ± 0.77) and uterine horns (6.25 ± 1.09 vs 3.39 ± 0.52) (^*^P < 0.05) (Figure 1A).

We then evaluated the percentage of Stro1^+^/CD44^+^ myometrial stem cells from uterocervix and uterine horns during different time points of pregnancy, to determine whether there were variations throughout the gestational process (Figure 1B). During the course of pregnancy, two different phases were identified: *expansion* (from day 0 until day 15), where the percentage of Stro1^+^/CD44^+^ myometrial stem cells increased significantly at day 15 (9.24 ± 0.85 in cervix versus 5.26 ± 0.76 in uterine horns) (^*^P-value < 0.05) and *involution*, from day 15 until the end of pregnancy (day 20), where the percentage of Stro1^+^/CD44^+^ myometrial stem cells decreased toward basal levels (5.24 ± 1.38 in cervix versus 4.06 ± 0.20 in uterine horns), although the differences were not statistically significant (Figure 1C). Consequently, it is unlikely that the uterus at the end of pregnancy, with all the humoral and mechanical impact of a full pregnancy and delivery, will promptly exhibit a nonpregnant profile. It is conceivable, though that, the myometrium will return to the nonpregnant status at the end of the puerperium period several days after delivery [2].

### Effect of low oxygen conditions and pregnant rat serum treatment on rat Stro1^+^/CD44^+^ myometrial stem cell proliferation

During pregnancy, the myometrium undergoes a profound physiological tissue remodeling, contributing to the dramatic expansion of the uterus. Therefore, although new myometrial cells might be developed from differentiated cells, they are more likely to be generated by primitive tissue-specific stem cells, which could contribute to the pregnancy-driven processes. Consequently, our main goal in this study was to evaluate the contribution of the myometrial stem cell population to uterine enlargement during pregnancy as well as to verify the hypoxia impact on the proliferation of myometrial stem cells.

**Figure 2.**
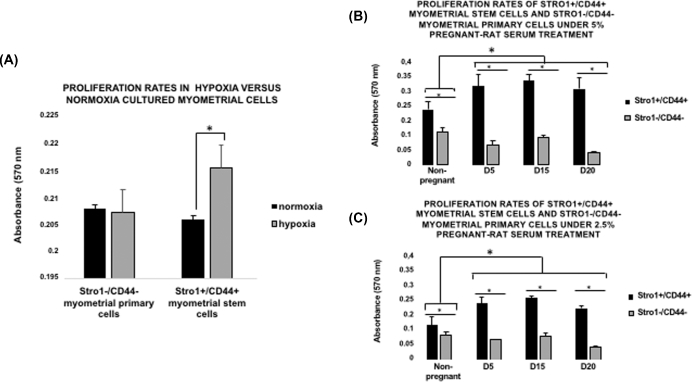
Comparison of cell proliferation rates under pregnant rat serum treatment and low oxygen conditions. (**A**) Proliferation rates of Stro1^+^/CD44^+^ myometrial stem cells under dose-dependent pregnant rat serum treatment, 5% (full concentration). Values are expressed as mean ± SEM for four independent animals per condition**. (B)** Proliferation rates of Stro1^+^/CD44^+^ myometrial stem cells under dose-dependent pregnant rat serum treatment 2.5% (half concentration). Values are expressed as mean ± SEM for four independent animals per condition**. (C)** Proliferation rates in hypoxia versus normoxia cultured myometrial cells: primary (–/–) and stem (+/+). Unpaired *t* test was used for statistical analysis ^*^P <0.05. Values are expressed as mean ± SEM for four independent animals per condition.

Recently, it has been demonstrated that low oxygen conditions promote the growth of many types of stem cells, including embryonic and mesenchymal stem cells [1,24,25]. Indeed, human Stro1^+^/CD44^+^ myometrial cells that exhibit myometrial stem cell-like properties can proliferate efficiently in vitro under hypoxic conditions (2% O_2_) [12]. Consistent with these observations, we observed that isolated Stro1^+^/CD44^+^ rat myometrial stem cells possessed a higher proliferative rate under hypoxic versus normoxic conditions (^*^P < 0.05) (Figure 2A). However, differences in isolated Stro1^−^/CD44^−^ myometrial primary cells were not statistically significant.

On the other hand, the endocrine and/or paracrine humoral factors produced during the course of a successful expanding pregnancy are probably responsible for the expansion of the myometrial stem cell population during the early- and mid-gestational phases and, further implicate these factors as being responsible for the distribution to the myometrium via circulation. To determine the potential effect of pregnant rat serum treatment on myometrial stem cell growth, Stro1^+^/CD44^+^ myometrial cells were employed and cultured in the absence or presence of 5% (full concentration) or 2.5% (half concentration) rat serum collected from days 5, 15, and 20 of normal timed pregnancy. In addition, nonpregnant serum and Stro1^−^/CD44^−^ myometrial primary cells were used under the same conditions as negative controls of the technique.

Interestingly, we noticed that Stro1^+^/CD44^+^ rat myometrial stem cells, cultured in the presence of 5% rat serum (full concentration), possessed a significantly higher proliferative rate than Stro1^−^/CD44^−^ myometrial primary cells in all the cases (^*^P < 0.05). Furthermore, statistical differences were found when we compare between pregnancy and nonpregnancy groups (^*^P < 0.05). However, differences in isolated Stro1^+^/CD44^+^ myometrial stem cells and Stro1^−^/CD44^−^ myometrial primary cells during diverse pregnancy days were not statistically significant (Figure 2B). Also, Stro1^+^/CD44^+^ myometrial stem cells treated with 2.5% (half concentration) rat serum showed significantly higher proliferation rates than Stro1^−^/CD44^−^ myometrial primary cells in all the cases (^*^P < 0.05). Moreover, statistical differences were found when we compare between pregnancy and nonpregnancy groups (^*^P < 0.05). However, differences in isolated Stro1^+^/CD44^+^ myometrial stem cells and Stro1^−^/CD44^−^ myometrial primary cells during diverse pregnancy days were not statistically significant (Figure 2C). In summary, this assay demonstrated that Stro1^+^/CD44^+^ myometrial stem cells respond efficiently to physiological cues during the uterine expansion (mid-gestational phase) and return to normal basal levels following delivery as result of endocrine and/or paracrine changes.

### Gene signature profile of hypoxia signaling in the Stro1^+^/CD44^+^ myometrial stem cells

It is well established that the rapid enlargement of the pregnant uterus causes increasing mechanical stretching of the uterine wall which outgrows the neovascularization process leading to focal hypoxia [1,14,15]. Since no significant differences between Stro1^−^/CD44^−^ myometrial primary cell population were found under hypoxic versus normoxic conditions, we felt that it was not prudent to analyze the gene signature profile of hypoxia signaling in this cell population. Nevertheless, based on the previous experiment, we proceeded to examine the regulatory link between oxygen availability and Stro1^+^/CD44^+^ myometrial stem cell function, to further explore the underlying mechanisms leading to the expansion of the Stro1^+^/CD44^+^ myometrial stem cell population during the early stages of pregnancy. A gene signature profile of hypoxia signaling was performed by Prime PCR arrays (BioRad). By using predefined criteria, *P*-value lower than 0.05, we identified a total of 37 upregulated versus 44 downregulated genes which were differentially expressed in the hypoxia versus normoxia-grown Stro1^+^/CD44^+^ myometrial stem cells (Table 1).

**Table 1.** List of genes up- and downregulated in the hypoxia versus normoxia-grown Stro-1^+^/CD44^+^ myometrial stem cells

Upregulated genes	Full gene name	Fold change
*Igfbp3*	Insulin like growth factor binding protein 3 precursor	46,650
*Mmp9*	Matrix metalloproteinase 9 precursor	17,915
*Plau*	Urokinase-type plasminogen activator precursor	7,568
*Mif*	Macrophage migration inhibitory factor	6,721
*Rbpjl*	Recombining binding protein suppressor of hairless-like protein	4,415
*Angptl4*	Angiopoietin-related protein 4	3,939
*Ndrg1*	Protein NDRG1	3,878
*Adm*	Adrenomedullin proadrenomedullin N-20 terminal peptide	3,302
*Ero1l*	ERO1-like protein alpha precursor	3,302
*Slc2a1*	Solute carrier family 2, facilitated glucose transporter member 1	2,467
*Pgk1*	Phosphoglycerate kinase 1	2,279
*Serpine1*	Plasminogen activator inhibitor 1 precursor	1,829
*Car9*	Carbonic anhydrase 9 precursor	1,712
*Slc2a3*	Solute carrier family 2, facilitated glucose transporter member 3	1,674
*Slc16a3*	Monocarboxylate transporter 4	1,606
*Pfkl*	6-Phosphofructokinase, liver type	1,563
*Ldha*	L-lactate dehydrogenase A chain	1,496
*Ankrd37*	Ankyrin repeat domain-containing protein 37	1,485
*Pgf*	Placental growth factor	1,453
*Gpi*	Glucose-6-phosphate isomerase	1,445
*Pdk1*	Pyruvate dehydrogenase kinase, isozyme 1 precursor	1,435
*Tpi1*	Triosephosphate isomerase 1	1,423
*Jmjd6*	Bifunctional arginine demethylase and lysyl-hydroxylase JMJD6	1,372
*Pfkfb3*	6-Phosphofructo-2-kinase/fructose-2, 6-bisphosphatase 3	1,246
*Hk2*	Hexokinase 2	1,223
*Pgam1*	Phosphoglycerate mutase 1	1,22
*Bnip3*	BCL2/adenovirus E1B interacting protein 3	1,186
*Pfkp*	6-Phosphofructokinase type C	1,163
*Egln1*	Egl nine homolog 1	1,106
*Blm*	Uncharacterized protein	1,096
*Fos*	Proto-oncogene c-Fos	1,072
*P4hb*	Protein disulfide-isomerase	1,071
*Aldoa*	Fructose-bisphosphate aldolase A	1,061
*Pim1*	Serine/threonine-protein kinase pim-1	1,057
*Ier3*	Immediate early response 3	1,050
*Ddit4*	DNA damage inducible transcript 4 protein	1,032
*Gys1*	Glycogen [starch] synthase, muscle	1,022
*Txnip*	Thioredoxin interacting protein	–4,769
*Btg1*	Protein BTG1	–2,653
*Edn1*	Endothelin 1	–2,272
*Eif4ebp1*	Eukaryotic translation initiation factor 4E binding protein 1	–2,168
*Egr1*	Early growth response protein 1	–2,075
*Tp53*	Cellular tumor antigen p53	–2,007
*Hif3a*	Hypoxia inducible factor 3-alpha	–2,001
*Odc1*	Ornithine decarboxylase 1	–1,998
*Apex1*	DNA-(apurinic or apyrimidinic site) lyase DNA-(apurinic or apyrimidinic site) lyase, mitochondrial	–1,892
*B2m*	Beta-2-microglobulin	–1,785
*ctsa*	Lysosomal protective protein precursor	–1,703
*Hmox1*	Heme oxygenase 1	–1,555
*Cops5*	COP9 signalosome complex subunit 5	–1,538
*Vdac1*	Voltage dependent anion-selective channel protein 1	–1,523
*Usf2*	Upstream stimulatory factor 2	–1,513
*Eno1*	Alpha-enolase	–1,500
*Hif1a*	Hypoxia inducible factor 1 alpha	–1,488
*Cdkn2a*	Cyclin dependent kinase inhibitor 2A	–1,455
*Pfkfb4*	6-Phosphofructo-2-kinase/fructose-2, 6-bisphosphatase 4	–1,443
*Lox*	Protein-lysine 6-oxidase	–1,425
*Mxi1*	Max interacting protein 1	–1,413
*Adora2b*	Adenosine A2b receptor	–1,409
*Vegfa*	Vascular endothelial growth factor A	–1,409
*Met*	Hepatocyte growth factor receptor precursor	–1,353
*Egln2*	Egl nine homolog 2	–1,348
*Nfkb1*	Nuclear factor NF-kappa-B p105 subunit nuclear factor NF-kappa-B p50 subunit	–1,330
*Dnajc5*	DnaJ homolog subfamily C member 5	–1,317
*Anxa2*	Annexin A2	–1,294
*Actb*	Actin, cytoplasmic 1 Actin, cytoplasmic 1, N-terminally processed	–1,263
*Pgm2*	Phosphoglucomutase 2	–1,251
*Arnt*	Aryl hydrocarbon receptor nuclear translocator	–1,228
*Bnip3l*	BCL2/adenovirus E1B 19 kDa protein-interacting protein 3-like	–1,214
*Ruvbl2*	ruvB-like 2	–1,212
*Hsp90ab1*	Heat shock protein HSP 90-beta	–1,211
*Lgals3*	Galectin 3	–1,136
*Map3k1*	Mitogen-activated protein kinase kinase kinase 1	–1,115
*Hif1an*	Hypoxia inducible factor 1 alpha inhibitor	–1,098
*P4ha1*	Prolyl 4-hydroxylase subunit alpha 1	–1,081
*Per1*	Period circadian protein homolog 1	–1,064
*Bhlhe40*	Class E basic helix-loop-helix protein 40	–1,043
*Gusb*	Beta-glucuronidase precursor	–1,038
*Tfrc*	Transferrin receptor protein 1	–1,025
*Nampt*	Nicotinamide phosphoribosyltransferase	–1,017
*Ccng2*	Cyclin G2	–1,008

Validation of the results obtained by the PCR array was performed by real-time PCR for *Igfbp3, Mmp9, Plau, Btg1, Edn1, Eif4ebp1, Egr1*, and *Tp53*. Consistent with the PrimePCR array, expression levels of *Igfbp3, Mmp9*, and *Plau* were upregulated in the hypoxia versus normoxia-grown Stro1^+^/CD44^+^ myometrial stem cells (Supplemental Figure S2). In addition, expression levels of *Btg1, Edn1, Eif4ebp1, Egr1*, and *Tp53* were shown to be downregulated in the hypoxia versus normoxia-grown Stro1^+^/CD44^+^ myometrial stem cells (Supplemental Figure S3).

### Functional analysis of the hypoxic gene signature in uterine expansion and remodeling

**Figure 3.**
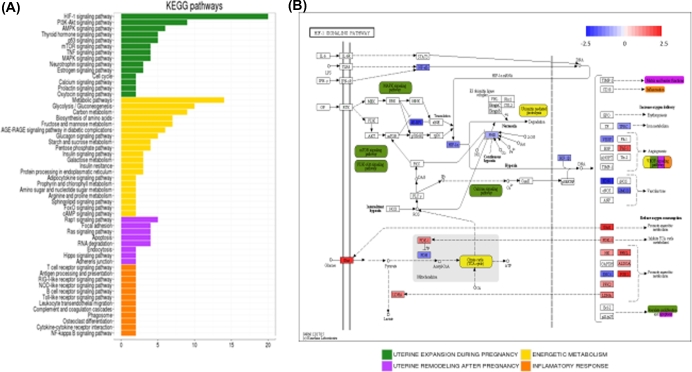
Functional meaning of hypoxia signature in uterine expansion and remodeling during pregnancy. (**A**) Distribution of implicated functions based on KEGG pathways database, where pathways were represented in (y) axis and number of genes belonging to each pathway are detailed in the x axis. Pathways were grouped into four main categories: uterine expansion during pregnancy (green), energy metabolism (yellow), uterine remodeling after pregnancy (pink), and inflammatory response (orange). (**B)** HIF-1 signaling pathway diagram containing the fold change representation for the 20 genes belonging to this pathway. Other pathways and functions represented by the genes (described in HIF-1 pathway) are shown in the same color that are shown in A section of this figure.

The analysis of the implicated functions through the KEGG pathways database provided us interesting connections related to selected genes. Figure 3 shows the details about the biological process and interactions from up- and obtained by Prime PCR arrays. Functional annotation of the genes from the KEGG pathways database is detailed in Supplemental Table S3, where only pathways with at least two genes annotated were considered and grouped into four main categories: uterine expansion during pregnancy, uterine remodeling after pregnancy, energy metabolism, and inflammatory response (Figure 3A).

Hypoxia inducible factor 1 (HIF-1), a transcription factor that functions as a master regulator of oxygen homeostasis, was highlighted as the most representative pathway in the gene signature (Figure 3B). Here, we identified the main downregulated hypoxia genes in the upper part of the figure (blue), while the most characteristic upregulated genes were found in the lower part of the figure (red). Interestingly, the HIF-1 signaling pathway interacted with other represented pathways in the hypoxic gene signature such as the MAPK, mTOR, PI3K-AKt, and calcium signaling pathway, and were included as part of the uterine expansion category (green). Ubiquitin-mediated proteolysis and citrate cycle pathways, related to energy metabolism (yellow), were also associated with the HIF-1 signaling pathway, as well as matrix and barrier functions, vascular endothelial growth factor (VEGF) signaling and apoptosis (pink) from the uterine remodeling category or the inflammatory response (orange).

**Figure 4.**
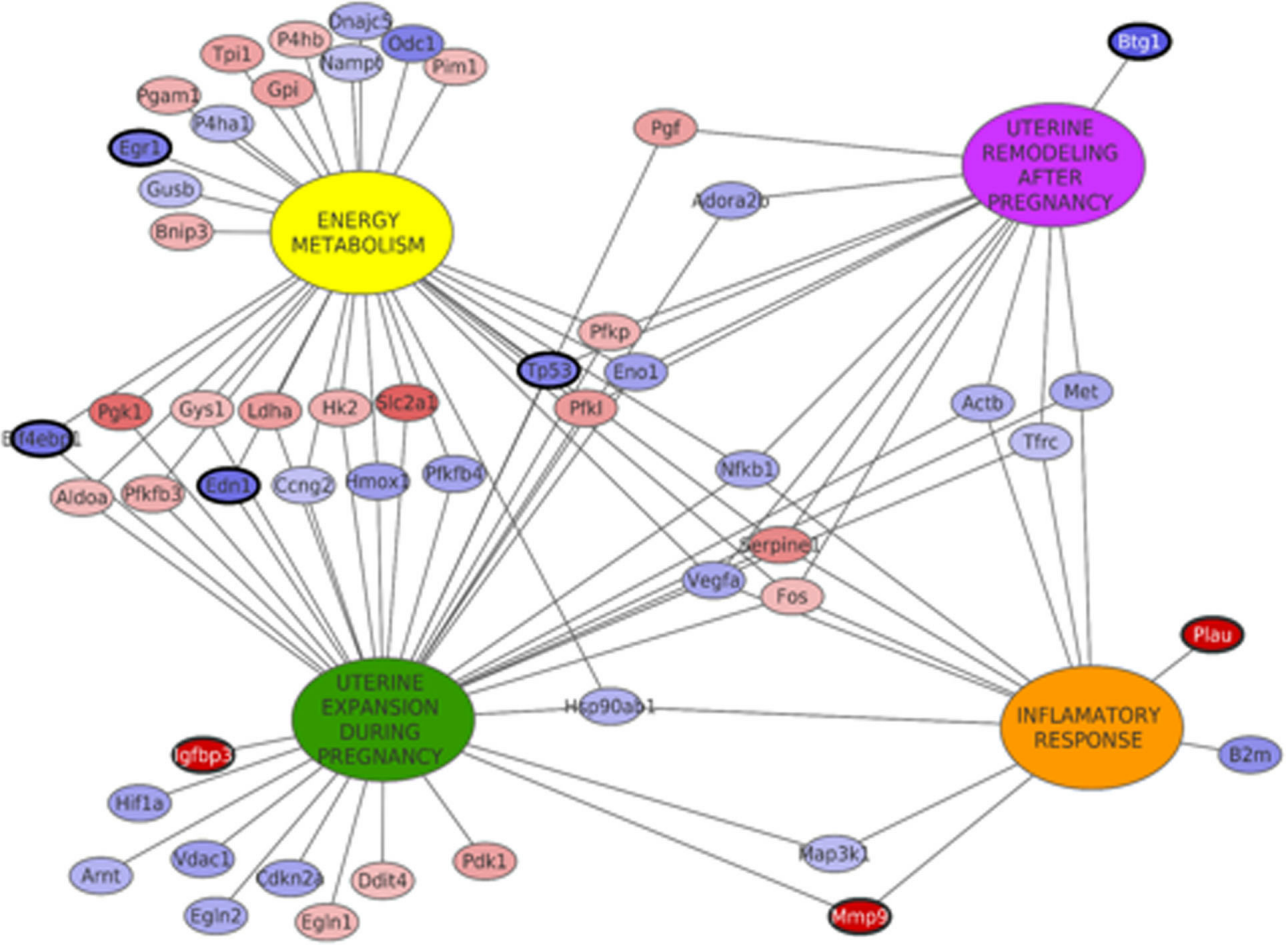
Network modeling of gene expression and functional relationship between uterine processes. Big nodes represent the main functions during the process and small nodes correspond to genes. Edges are linking genes belonging to functions. The model is representing the four designed functions and the genes that are shared by them. Genes in red are upregulated in hypoxia and genes in blue are downregulated in hypoxia. Bold circles are validated genes by RT-qPCR.

Finally, we used Cytoscape software to create a relationship network between functions and genes, which could augment our efforts in understanding the regulatory link between oxygen availability and Stro1^+^/CD44^+^ myometrial stem cell function during pregnancy (Figure 4). Big nodes represent the main categorical functions in the related process, whereas small spheres represent genes obtained by Prime PCR arrays. Analysis of this network highlighted two significant categories, uterine expansion and energy metabolism, with a great number of connections between both fractions. Moreover, all the functions were connected among them by genes that belong to more than one function. Specifically, four of these genes were shared by all functions: *Serpine1* and *Fos* (upregulated in hypoxia); *Nfkb1* and *Vegfa* (downregulated in hypoxia), as main regulators in the general process.

## Discussion

To date, increasing evidence indicates that myometrial regeneration and remodeling processes during pregnancy involve the myometrial stem cell population [1,26,27]. Indeed, putative myometrial stem and/or progenitor cells in humans [12,13] and rodents [18,19] have been recently identified, isolated, and characterized. In these previous studies, we confirmed the undifferentiated status of isolated Stro1^+^/CD44^+^ myometrial cells by the expression of stem cell markers OCT4, C-KIT, and DNMT3B, and the low expression of steroid receptors ERα and PR-A/PR-B. In addition, the expression of typical hematopoietic markers CD45 or endothelial markers like CD31 was barely detectable. Finally, their functional capability to form regenerated myometrial tissues from Stro1^+^/CD44^+^ myometrial stem cells was established in a unique xenotransplantation mouse model, where we demonstrated the ability to differentiate into myometrial cells by the expression of actin alpha-2 smooth muscle antigen and progesterone receptor [12,18,19].

The study presented here addresses the expression of Stro1 and CD44 cell surface markers in murine myometrium during various stages of pregnancy. Stro1 is a cell surface antigen that has shown robust enrichment for somatic stem cells, but not by committed hematopoietic progenitors [28,29]. CD44 is a cell surface glycoprotein involved in cell proliferation, differentiation, and migration [30]. Using Stro1/CD44 surface markers, we were able to isolate and quantify the myometrial stem cell population in the gestational and postpartum murine myometrium, an essential step toward understanding the involvement of putative stem cells in remodeling events. As shown in Figure 1A, it became immediately apparent that multiparity caused significant and seemingly permanent irreversible expansion of myometrial stem cells. Furthermore, the results presented here suggest that the elevated growth potential in the rat myometrium during pregnancy could be the result of increased percentages of Stro1^+^/CD44^+^ myometrial stem cells in the uterine horns, in addition to hyperplasia and hypertrophy processes. In addition, the cervix could be an important reservoir generating new myometrial cells to support the pregnancy in the initial phase in response to specific stimuli like steroidal hormone input.

Estrogen (E2) and progesterone (P4) may play an important role in the upregulation and proliferation of Stro1^+^/CD44^+^ myometrial stem cells in pregnant myometrium [13]. However, because Stro1^+^/CD44^+^ myometrial stem cells underexpress E2 and P4 receptors [12], it is possible that differentiated myometrial cells may produce unknown factors in response to hormonal signaling, and thus indirectly promote the proliferation of Stro1^+^/CD44^+^ myometrial stem cells in a paracrine manner. Interestingly, when we evaluated the effect of pregnant rat serum treatment on the rat Stro1^+^/CD44^+^ myometrial stem cell proliferation, we observed that cells efficiently responded to these physiological stimuli (Figure 2A and B). Further studies, however, are needed to identify which factors are involved in this process.

In addition to hormone signaling, hypoxia is thought to be important for the maintenance of stemness and suppression of cell senescence in stem cells [1]. Many studies published to date have revealed strong regulatory links between limited oxygen availability, proliferation, differentiation, and function of trophoblasts and placenta during pregnancy [24,25,31]. Recent reports in animal research suggest that during pregnancy, the mechanical stretching of the uterine wall, a sequence critical in determining the success of a pregnancy, could induce low oxygen conditions, [32,33]. Specifically, the tuberous sclerosis tumor suppressor proteins TSC2, in carrier Eker rats, form a molecular complex that functions to integrate the cellular response to growth factor, nutrient, and oxygen availability [34]. Under hypoxic conditions, cells rapidly activate a variety of adaptive mechanisms that limit energy expenditure through the inhibition of energy-intensive processes including protein translation [35,36], as shown in Figure 3B. Moreover, genetic studies have shown the tuberous sclerosis tumor suppressors TSC2 and the REDD1 protein to be essential for hypoxia regulation of TORC1 activity in mammalian cells [37]. Consistent with these observations, Stro1^+^/CD44^+^ myometrial stem cells are well suited for in vitro growth in a hypoxic environment, but proliferated poorly under normoxic conditions (Figure 2C). These data are in accordance with recent publications demonstrating that human myometrial stem cells preferentially expand *in vitro* under 2% oxygen [12,17]. Nevertheless, further studies are needed to elucidate the exact role of myometrial stem cells in noncarrier TSC2 rats and whether disruption in normal stem cell proliferation may result in obstetrical disorders.

Finally, gene signature profiles of the hypoxia signaling pathway allowed us to identify 37 upregulated versus 44 downregulated genes which were differentially expressed in the hypoxia versus normoxia-grown Stro1^+^/CD44^+^ myometrial stem cells, providing an alternative approach inferring molecular mechanisms for complex functions such as energy metabolism, inflammatory response, and uterine expansion and/or remodeling.

Interestingly, HIF-1 was highlighted as the most representative pathway in the gene signature, acting as a master regulator of numerous hypoxia inducible genes under hypoxic conditions [38]. The target genes of HIF-1 encode proteins that increase O_2_ delivery and mediate adaptive responses to O_2_ deprivation. However, despite its name, HIF-1 is induced not only in response to reduced oxygen availability, but also by other stimulants, such as nitric oxide, and various growth factors.

To date, more than 100 genes have been identified as the transcriptional target of *HIF-1*. Among them, vascular endothelial growth factor (*Vegfa*) is the most potent endothelial-specific mitogen, which recruits endothelial cells into hypoxic foci and avascular area and stimulates their proliferation. Thus, HIF and *Vegfa* signaling is essential for the maintenance of the vascular density and oxygen supply in hypoxic tissue. Similarly, the expression of *Serpine1*, an important regulator of inflammation and angiogenesis processes, is induced by hypoxia at the transcriptional level, via the *HIF-1* and cellular Fos (*c-Fos*) [39,40]. Moreover, it has also reported that *Nfkb1* is directly regulated by *HIF-1a*, being both the key regulators linked with hypoxia and inflammation process [40].

Specifically, the aforementioned genes, *Vegfa, Serpine1, Nfkb1*, and *Fos*, which were shared by all functions, were also considered key genes in the pregnancy process during hypoxic conditions as shown in Figure 4. For many years, it has been suggested that the establishment of pregnancy requires changes in the uterus that allow for attachment and implantation. Given the fact that the uterine transcriptional profile during early development resembles a proinflammatory response, it is possible that the transcription factor, nuclear factor kappa B subunit 1(*NFKB1*), is involved in the establishment of pregnancy [41]. Moreover, Fos genes and proteins have been implicated as regulators of cell proliferation, differentiation, and transformation with increased expression catalyzed by a variety of stimuli, including growth factors, cytokines, hormones, stress, and cell injury [42]. In addition, during pregnancy, *VEGF-A* is also essential for the proliferation of trophoblasts, the development of embryonic vasculature, and the growth of maternal and fetal blood cells in utero [43], as well as the restoration of the oxygen supply to tissues when blood circulation is inadequate such as in hypoxic conditions [44]. However, the mammalian uterus undergoes drastic tissue remodeling not only during pregnancy, but also through the estrous cycle and implantation. As such, tissue remodeling requires a fine-tuned balance between levels of proteases and their cognate inhibitors. Specifically, *SERPINE1* was demonstrated to be present in human and murine uteri during implantation and tissue remodeling [45,46].

In conclusion, our data support the concept that rat Stro1^+^/CD44^+^ myometrial stem cells contribute to uterine enlargement during pregnancy. Since these cells preferentially grow under low oxygen conditions, we propose that pregnancy-induced mechanical stretching results in hypoxia, that in turn promotes stem cell proliferation and further uterine enlargement, positively contributing to a successful pregnancy. Further studies, including gene expression profiles during the different physiologic states of pregnancy, could provide an experimental approach in better understanding the biology of human myometrium. Future studies will also afford the opportunity to elucidate the exact role myometrial stem cells play in ensuring a healthy pregnancy and reveal their role in pathological conditions, such as preterm labor.

## Supplemental data

Supplementary data are available at *BIOLRE* online.

**Supplemental Figure S1.** Flow cytometric analysis of isolated myometrial cells displaying the negative expression of (A) hematopoietic stem cell (CD45) and (B) endothelial (CD31) markers.

**Supplemental Figure S2.** Gene validation of the upregulated *Igfbp3, Mmp9*, and *Plau* in the hypoxia versus normoxia-grown Stro1^+^/CD44^+^ myometrial stem cells. (A) Expression level of *Igfbp3*, (B) expression level of *Mmp9*, (C) expression level of *Plau*. Three biological samples from hypoxia versus normoxia-grown Stro1^+^/CD44^+^ myometrial stem cell samples were subjected to RNA isolation, cDNA synthesis, and q-PCR analysis. Each experiment was conducted in replicates. ^*^P < 0.05 compared with the respective control.

**Supplemental Figure S3.** Gene validation of the downregulated *Btg1, Edn1, Eif4ebp1, Egr1*, and *Tp53* in the hypoxia versus normoxia-grown Stro1^+^/CD44^+^ myometrial stem cells. (A) Expression level of *Btg1, (*B) expression level of *Edn1*, (C) expression level of *Eif4ebp1*, (D) expression level of *Egr1*, and (E) expression level of *Tp53.* Three biological samples from hypoxia versus normoxia-grown Stro1^+^/CD44^+^ myometrial stem cell samples were subjected to RNA isolation, cDNA synthesis, and q-PCR analysis. Each experiment was conducted in replicates. ^*^P < 0.05 compared with the respective control.

**Supplemental Table S1.** List of antibodies used for immunophenotypic analysis and cell isolation.

**Supplemental Table S2.** List of rat primers sequences used for gene expression validation.

**Supplemental Table S3.** Functional gene annotation in KEGG-containing KEGG term ID and pathway name, number of genes from the annotated signature, name of the genes, number of total genes from the genome in the pathway, and briefly description of the pathway. (A) Uterine expansion during pregnancy. (B) Energy metabolism. (C) Uterine remodeling after pregnancy. (D) Inflammatory response.

Supplemental material
