# Supplementary material for: Role of Stro1+/CD44+ stem cells in myometrial physiology and uterine remodeling during pregnancy
Source: Biol Reprod. 2016 Dec 23;96(1):70–80. doi: 10.1095/biolreprod.116.143461 (PMC5803774; doi:10.1095/biolreprod.116.143461)
Supplement: Supplemental material [file bio143461_supp.zip › Supplemental Table1.pdf]

SUPPLEMENTAL INFORMATION

Supplemental Table 1. List of primers sequences used for gene expression validation in rat.

Gene	Primer sequence	Assay
Insulin-like growth factor binding protein-3 (Igfbp3)	F: CAGGCAGAATGTCTCAAGAT	q-PCR
	R: AATACAGGGTGTGAGGAGA	q-PCR
Matrix metallopeptidase-9 (Mmp9)	F: ACTAAAGGTCGCTCGGAT	q-PCR
	R: AGTAGGACAGAAGCCATACA	q-PCR
Plasminogen-activator urokinase (Plau)	F: TAAGAGGTCCTCCTGAATCTCC	q-PCR
	R: CAAGCAGCCTCACTACTATGG	q-PCR
Protein BTG1 (Btg1)	F: GAAGATCTGGCACAAGATAGAG	q-PCR
	R: GGACTCACAGGCTACTTAAATC	q-PCR
Endothelin-1 (Edn1)	F: AAGCTGGGAAAGAAGTGTATC	q-PCR
	R: CAACTCGAAAGGAGGTCTTG	q-PCR
Eukaryotic translation initiation factor 4E binding protein1(Eif4bp1)	F: CGGAATGATCTGGCAATTCTA	q-PCR
	R: AGA AGT AAA GGG TGG CTT TC	q-PCR
Early growth response protein 1 (Egr1)	F: GTTTGCCAGGAGTGATGAA	q-PCR
	R: GGTAGGAAGAGAGGGAAGAG	q-PCR
Tumor protein p53 (Tp53)	F: GAAATCCTATCCGGTCAGTTG	q-PCR
	R: GTATGAGGGCCCAAGATAGA	q-PCR
18s	F: CACGGACAGGATTGACAGATT	q-PCR
	R: GAGTCTCGTTCGTTATCGGAATTA	q-PCR
