# Supplementary material for: Role of Stro1+/CD44+ stem cells in myometrial physiology and uterine remodeling during pregnancy
Source: Biol Reprod. 2016 Dec 23;96(1):70–80. doi: 10.1095/biolreprod.116.143461 (PMC5803774; doi:10.1095/biolreprod.116.143461)
Supplement: Supplemental material [file bio143461_supp.zip › Supplemental Table2.pdf]

Supplemental Table2. Functional gene annotation in KEGG.

A) Uterine expansion during pregnancy.

ID	NAME	N.GENES	GENES	TOTAL	DESCRIPTION
rno04066	HIF-1 signaling pathway	20	*Aldoa; Arnt; Edn1; Egln1; Egln2; Eif4ebp1; Eno1; Hif1a; Hk2; Hmox1; Ldha; Nfkb1; Pdk1; Pfkfb3; Pfkl; Pgk1; Serpine1; Slc2a1; Tfrc; Vegfa*	109	Hypoxia-inducible factor 1 (HIF-1) is a transcription factor that functions as a master regulator of oxygen homeostasis. The target genes of HIF-1 encode proteins that increase O2 delivery and mediate adaptive responses to O2 deprivation. HIF-1 is induced not only in response to reduced oxygen availability but also by other stimulants, such as nitric oxide, or various growth factors.
rno04151	PI3K-Akt signaling pathway	9	*Ddit4; Eif4ebp1; Gys1; Hsp90ab1; Met; Nfkb1; Pgf; Tp53; Vegfa*	336	The phosphatidylinositol 3' -kinase(PI3K)-Akt signaling pathway is activated by many types of cellular stimuli or toxic insults and regulates fundamental cellular functions such as transcription, translation, proliferation, growth, and survival.
rno04152	AMPK signaling pathway	6	*Eif4ebp1; Gys1; Pfkfb3; Pfkfb4; Pfkl; Pfkp*	129	AMP-activated protein kinase (AMPK) is a serine threonine kinase that is highly conserved through evolution. AMPK system acts as a sensor of cellular energy status. It is activated by increases in the cellular AMP: ATP ratio caused by metabolic stresses that either interfere with ATP production or that accelerate ATP consumption.
rno04919	Thyroid hormone signaling pathway	5	*Actb; Hif1a; Pfkp; Slc2a1; Tp53*	119	The thyroid hormones (THs) are important regulators of growth, development and metabolism. The action of TH is mainly mediated by T3 (3,5,3'-triiodo-L-thyronine). Thyroid hormones, L-thyroxine (T4) and T3 enter the cell through transporter proteins.
rno04115	p53 signaling pathway	5	*Ccng2; Cdkn2a; Igfbp3; Serpine1; Tp53*	71	p53 activation is induced by a number of stress signals, including DNA damage, oxidative stress and activated oncogenes. The p53 protein is employed as a transcriptional activator of p53-regulated genes. This results in three major outputs; cell cycle arrest, cellular senescence or apoptosis. Other p53-regulated gene functions communicate with adjacent cells, repair the damaged DNA or set up positive and negative feedback loops that enhance or attenuate the functions of the p53 protein and integrate these stress responses with other signal transduction pathways.
rno04150	mTOR signaling pathway	4	*Ddit4; Eif4ebp1; Hif1a; Vegfa*	64	The mechanistic target of rapamycin (mTOR), is a protein that in humans is encoded by the MTOR gene. MTOR is a serine/threonine protein kinase that regulates cell growth, cell proliferation, cell motility, cell survival, protein synthesis, autophagy, transcription. MTOR belongs to the phosphatidylinositol 3-kinase-related kinase protein family, and fibrosis.
rno04668	TNF signaling pathway	4	*Edn1; Fos; Mmp9; Nfkb1*	110	Tumor necrosis factor (TNF), as a critical cytokine, can induce a wide range of intracellular signal pathways including apoptosis and cell survival as well as inflammation and immunity.
rno04010	MAPK signaling pathway	4	*Fos; Map3k1; Nfkb1; Tp53*	259	The mitogen-activated protein kinase (MAPK) cascade is a highly conserved module involved in various cellular functions, including cell proliferation, differentiation and migration.
rno04722	Neurotrophin signaling pathway	3	*Map3k1; Nfkb1; Tp53*	126	Neurotrophins are a family of trophic factors involved in cell differentiation and survival. Neurotrophin/Trk signaling is regulated by connecting a variety of intracellular signaling cascades, which include MAPK pathway, PI-3 kinase pathway, and PLC pathway, transmitting positive signals like enhanced survival and growth.
rno04915	Estrogen signaling pathway	3	*Fos; Hsp90ab1; Mmp9*	96	Estrogens are steroid hormones that regulate physiological processes in mammals, including reproduction, cellular homeostasis, and behavior. Estrogen mediates its cellular actions through two signaling pathways classified as "nuclear-initiated steroid signaling" and "membrane-initiated steroid signaling". In the "nuclear" pathway, estrogen binds either ERalpha or ERbeta, which in turn translocates to the nucleus, binds DNA at ERE elements and activates the expression of ERE-dependent genes. In "membrane" pathway, Estrogen can exert its actions through a subpopulation of ER at the plasma membrane (mER) or novel G-protein coupled E2 receptors (GPER). Upon activation of these receptors various signaling pathways are rapidly activated and ultimately influence downstream transcription factors.
rno04110	Cell cycle	2	*Cdkn2a; Tp53*	127	Mitotic cell cycle progression is accomplished through a reproducible sequence of events, DNA replication (S phase) and mitosis (M phase) separated temporally by gaps known as G1 and G2 phases. Cyclin-dependent kinases (CDKs) are key regulatory enzymes, each consisting of a catalytic CDK subunit and an activating cyclin subunit. CDKs regulate the cell's progression through the phases of the cell cycle by modulating the activity of key substrates.
rno04020	Calcium signaling pathway	2	*Adora2b; Vdac1*	187	Ca2+ that enters the cell from the outside is a principal source of signal Ca2+. Entry of Ca2+ is driven by the presence of a large electrochemical gradient across the plasma membrane. Cells use this external source of signal Ca2+ by activating various entry channels with widely different properties.
rno04917	Prolactin signaling pathway	2	*Fos; Nfkb1*	76	Prolactin (PRL) is a polypeptide hormone known to be involved in a wide range of biological functions including osmoregulation, lactation, reproduction, growth and development, endocrinology and metabolism, brain and behavior, and immunomodulation.
rno04921	Oxytocin signaling pathway	2	*Actb; Fos*	160	Oxytocin (OT) exerts a wide variety of central and peripheral effects. However, its best-known and most well-established roles are stimulation of uterine contractions during parturition and milk release during lactation. The actions of OT are all mediated by one type of OT receptor (OTR). This is a transmembrane receptor belonging to the G-protein-coupled receptor superfamily. The main signaling pathway is the Gq/PLC/Ins3 pathway, but the MAPK and the RhoA/Rho kinase pathways are also activated, contributing to increased prostaglandin production and direct contractile effect on myometrial cells.

B) Energy Metabolism.

ID	NAME	N.GENES	GENES	TOTAL	DESCRIPTION
rno01100	Metabolic pathways	14	*Aldoa; Eno1; Gpi; Gusb; Hk2; Ldha; Nampt; Odc1; P4ha1; Pfkl; Pfkp; Pgam1; Pgk1; Tpi1*	1304	Metabolic pathway is a linked series of chemical reactions occurring within a cell. The reactants, products, and intermediates of an enzymatic reaction are known as metabolites, which are modified by a sequence of chemical reactions catalyzed by enzymes.
rna00010	Glycolysis / Gluconeogenesis	10	*Aldoa; Eno1; Gpi; Hk2; Ldha; Pfkl; Pfkp; Pgam1; Pgk1; Tpi1*	70	Glycolysis is the process of converting glucose into pyruvate and generating small amounts of ATP (energy) and NADH (reducing power). Gluconeogenesis is a synthesis pathway of glucose from noncarbohydrate precursors. It is essentially a reversal of glycolysis with minor variations of alternative paths.
rno01200	Carbon metabolism	9	*Aldoa; Eno1; Gpi; Hk2; Pfkl; Pfkp; Pgam1; Pgk1; Tpi1*	121	Carbon metabolism is the most basic aspect of life. The map contains carbon utilization pathways of glycolysis, pentose phosphate pathway, and citrate cycle, and six known carbon fixation pathways as well as some pathways of methane metabolism.
rno01230	Biosynthesis of amino acids	7	*Aldoa; Eno1; Pfkl; Pfkp; Pgam1; Pgk1; Tpi1*	83	This map presents a modular architecture of the biosynthesis pathways of twenty amino acids, which may be viewed as consisting of the core part and its extensions. The core part is the KEGG module for conversion of three-carbon compounds from glyceraldehyde-3P to pyruvate, together with the pathways around serine and glycine.
rno00051	Fructose and mannose metabolism	7	*Aldoa; Hk2; Pfkfb3; Pfkfb4; Pfkl; Pfkp; Tpi1*	38	This pathway utilises fructose, a hexose and fructose-6-phosphate, a glycolysis intermediate for the production of important nucleotide sugars such as GDP-D-mannose and GDP-L-fucose. These compounds are essential substrates for glycosylphosphatidylinositol (GPI) anchor biosynthesis and synthesis of the anchors for N-glycans.
rno04933	AGE-RAGE signaling pathway in diabetic complications	6	*Edn1; Egr1; Nfkb1; Pim1; Serpine1; Vegfa*	104	Advanced glycation end products (AGEs) are a complex group of compounds produced through the non-enzymatic glycation and oxidation of proteins, lipids and nucleic acids, primarily due to aging and under certain pathologic condition such as hyperglycemia.
rno04922	Glucagon signaling pathway	5	*Gys1; Ldha; Pfkl; Pgam1; Slc2a1*	102	Glucagon is conventionally regarded as a counterregulatory hormone for insulin and plays a critical anti-hypoglycemic role by maintaining glucose homeostasis in both animals and humans.
rno00500	Starch and sucrose metabolism	4	*Gpi; Gusb; Gys1; Hk2*	53	Sucrose metabolism plays pivotal roles in development, stress response, and yield formation, mainly by generating a range of sugars as metabolites to fuel growth and synthesize essential compounds and as signals to regulate expression of microRNAs, transcription factors, and other genes and for crosstalk with hormonal, oxidative, and defense signaling.
rno00030	Pentose phosphate pathway	4	*Aldoa; Gpi; Pfkl; Pfkp*	29	The pentose phosphate pathway is a process of glucose turnover that produces NADPH as reducing equivalents and pentoses as essential parts of nucleotides.
rno04910	Insulin signaling pathway	3	*Eif4ebp1; Gys1; Hk2*	140	Biochemical pathway by which insulin increases the uptake of glucose into fat and muscle cells and reduces the synthesis of glucose in the liver and hence is involved in maintaining glucose homeostasis. This pathway is also influenced by fed versus fasting states, stress levels, and a variety of other hormones.
rno00052	Galactose metabolism	3	*Hk2; Pfkl; Pfkp*	32	Galactose metabolism, which converts galactose into glucose, is carried out by the three principal enzymes in a mechanism known as the Leloir pathway. The enzymes are listed in the order of the metabolic pathway: galactokinase (GALK), galactose-1-phosphate uridyltransferase (GALT), and UDP-galactose-4'-epimerase (GALE).
rno04931	Insulin resistance	3	*Gys1; Nfkb1; Slc2a1*	111	Insulin resistance is a condition where cells become resistant to the effects of insulin due to (a) increased phosphorylation of insulin receptor substrate protein, (b) increased IRS-1 proteasome degradation via mTOR signaling pathway, (c) decreased activation of signaling molecules including PI3K and AKT, (d) increase in activity of phosphatases including PTPs, PTEN, and PP2A.
rno04141	Protein processing in endoplasmatic reticulum	3	*Dnajc5; Hsp90ab1; P4hb*	168	The endoplasmic reticulum (ER) is a subcellular organelle where proteins are folded with the help of lumenal chaperones. Correctly folded proteins are packaged into transport vesicles that shuttle them to the Golgi complex. Misfolded proteins are retained within the ER lumen in complex with molecular chaperones.
rno04920	Adipocytokine signaling pathway	2	*Nfkb1; Slc2a1*	75	Increased adipocyte volume and number are positively correlated with leptin production, and negatively correlated with production of adiponectin.
rno00860	Prophyrin and chlorophyll metabolism	2	*Gusb; Hmox1*	41	Porphyrin and chlorophyll metabolism
rno00520	Amino sugar and nucleotide sugar metabolism	2	*Gpi; Hk2*	48	Amino sugar and nucleotide sugar metabolism
rno00330	Arginine and proline metabolism	2	*Odc1; P4ha1*	51	Arginine and proline metabolism
rno04071	Sphingolipid signaling pathway	2	*Nfkb1; Tp53*	124	Sphingomyelin (SM) and its metabolic products are now known to have second messenger functions in a variety of cellular signaling pathways.
rno04068	FoxO signaling pathway	2	*Bnip3; Ccng2*	136	The forkhead box O (FOXO) family of transcription factors regulates the expression of genes in cellular physiological events including apoptosis, cell-cycle control, glucose metabolism, oxidative stress resistance, and longevity.
rno04024	cAMP signaling pathway	2	*Fos; Nfkb1*	196	cAMP is one of the most common and universal second messengers, and its formation is promoted by adenylyl cyclase (AC) activation after ligation of G protein-coupled receptors (GPCRs) by ligands including hormones, neurotransmitters, and other signaling molecules. cAMP regulates pivotal physiologic processes including metabolism, secretion, calcium homeostasis, muscle contraction, cell fate, and gene transcription.

C) Uterine remodeling after pregnancy.

ID	NAME	N.GENES	GENES	TOTAL	DESCRIPTION
rno04015	Rap1 signaling pathway	5	*Actb; Adora2b; Met; Pgf; Vegfa*	216	Rap1 is a small GTPase that controls diverse processes, such as cell adhesion, cell-cell junction formation and cell polarity. Rap1 also regulates MAP kinase (MAPK) activity in a manner highly dependent on the context of cell types.
rno04510	Focal adhesion	4	*Actb; Met; Pgf; Vegfa*	206	Cell-matrix adhesions play essential roles in important biological processes including cell motility, cell proliferation, cell differentiation, regulation of gene expression and cell survival.
rno04014	Ras signaling pathway	4	*Met; Nfkb1; Pgf; Vegfa*	232	The Ras proteins are GTPases that function as molecular switches for signaling pathways regulating cell proliferation, survival, growth, migration, differentiation or cytoskeletal dynamism.
rno04210	Apoptosis	4	*Actb; Fos; Nfkb1; Tp53*	141	Apoptosis is a genetically programmed process for the elimination of damaged or redundant cells by activation of caspases (aspartate-specific cysteine proteases). The onset of apoptosis is controlled by numerous interrelating processes.
rno03018	RNA degradation	4	*Btg1; Eno1; Pfkl; Pfkp*	82	The correct processing, quality control and turnover of cellular RNA molecules. It is critical in many aspects for the expression of genetic information.
rno04390	Hippo signaling pathway	2	*Actb; Serpine1*	156	Hippo signaling is an evolutionarily conserved signaling pathway that controls organ size from flies to humans.
rno04144	Endocytosis	2	*Met; Tfrc*	288	Endocytosis is a mechanism for cells to remove ligands, nutrients, and plasma membrane (PM) proteins, and lipids from the cell surface, bringing them into the cell interior.
rno04520	Adherens junction	2	*Actb; Met*	74	Cell-cell adherens junctions (AJs), the most common type of intercellular adhesions, are important for maintaining tissue architecture and cell polarity and can limit cell movement and proliferation.

D) Inflammatory Response.

ID	NAME	N.GENES	GENES	TOTAL	DESCRIPTION
rno04660	T cell receptor signaling pathway	2	*Fos; Nfkb1*	109	Activation of T lymphocytes is a key event for an efficient response of the immune system. It requires the involvement of the T-cell receptor (TCR) as well as costimulatory molecules, resulting in a series of signaling cascades which comprise an array of protein-tyrosine kinases, phosphatases, GTP-binding proteins and adaptor proteins that regulate generic and specialized functions, leading to T-cell proliferation, cytokine production and differentiation into effector cells.
rno04612	Antigen processing and presentation	2	*B2m; Hsp90ab1*	98	Antigen processing and presentation
rno04622	RIG-I-like receptor signaling pathway	2	*Map3k1; Nfkb1*	64	Specific families of pattern recognition receptors which are responsible for detecting viral pathogens and generating innate immune responses.
rno04621	NOD-like receptor signaling pathway	2	*Hsp90ab1; Nfkb1*	58	Specific families of pattern recognition receptors are responsible for detecting various pathogens and generating innate immune responses.
rno04662	B cell receptor signaling pathway	2	*Fos; Nfkb1*	74	B cells are an important component of adaptive immunity. They produce and secrete millions of different antibody molecules, each of which recognizes a different antigen.
rno04620	Toll-like receptor signaling pathway	2	*Fos; Nfkb1*	97	Specific families of pattern recognition receptors are responsible for detecting microbial pathogens and generating innate immune responses
rno04670	Leukocyte transendothelial migration	2	*Actb; Mmp9*	120	Leukocyte migaration from the blood into tissues is vital for immune surveillance and inflammation.
rno04610	Complement and coagulation cascades	2	*Plau; Serpine1*	82	The complement system is a proteolytic cascade in blood plasma and a mediator of innate immunity, a nonspecific defense mechanism against pathogens. There are three pathways of complement activation: the classical pathway, the lectin pathway, and the alternative pathway. All of these pathways generate a crucial enzymatic activity that, in turn, generates the effector molecules of complement.
rno04145	Phagosome	2	*Actb; Tfrc*	198	Phagocytosis is the process of taking in relatively large particles by a cell, and is a central mechanism in the tissue remodeling, inflammation, and defense against infectious agents.
rno04380	Osteoclast differentiation	2	*Fos; Nfkb1*	134	The osteoclasts, multinucleared cells originating from the hematopoietic monocyte-macrophage lineage, are responsible for bone resorption.
rno04060	Cytokine-cytokine receptor interaction	2	*Met; Vegfa*	221	Cytokines are soluble extracellular proteins or glycoproteins that are crucial intercellular regulators and mobilizers of cells engaged in innate as well as adaptive inflammatory host defenses, cell growth, differentiation, cell death, angiogenesis, and development and repair processes aimed at the restoration of homeostasis.
rno04064	NF-kappa B signaling pathway	2	*Nfkb1; Plau*	95	Nuclear factor-kappa B (NF-kappa B) is the generic name of a family of transcription factors that function as dimers and regulate genes involved in immunity, inflammation and cell survival.
