# Supplementary figures and images for: Role of Stro1+/CD44+ stem cells in myometrial physiology and uterine remodeling during pregnancy
Source: Biol Reprod. 2016 Dec 23;96(1):70–80. doi: 10.1095/biolreprod.116.143461 (PMC5803774; doi:10.1095/biolreprod.116.143461)

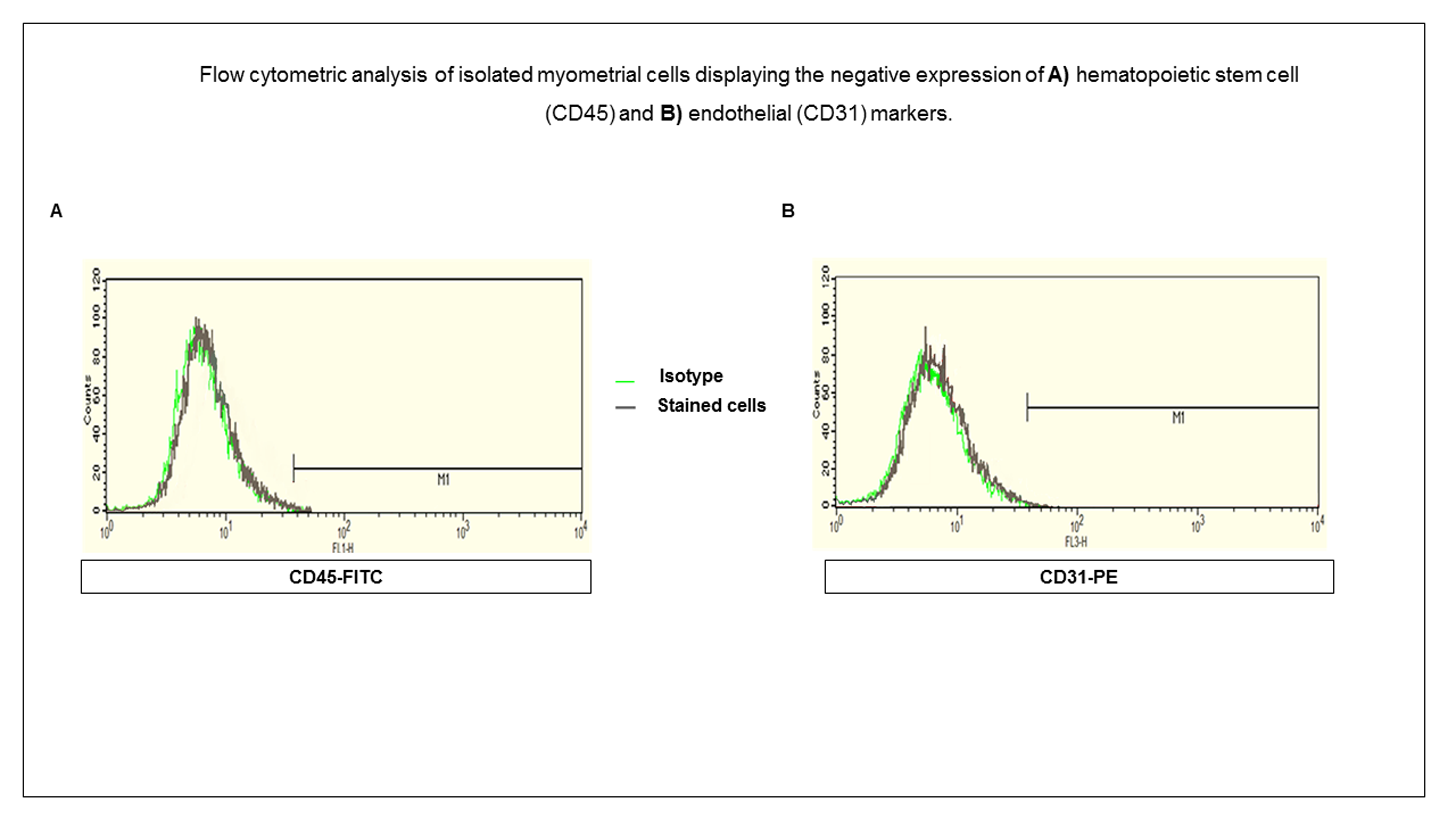

Supplement: Supplemental material [file bio143461_supp.zip › Supplemental Figure 1.tif]

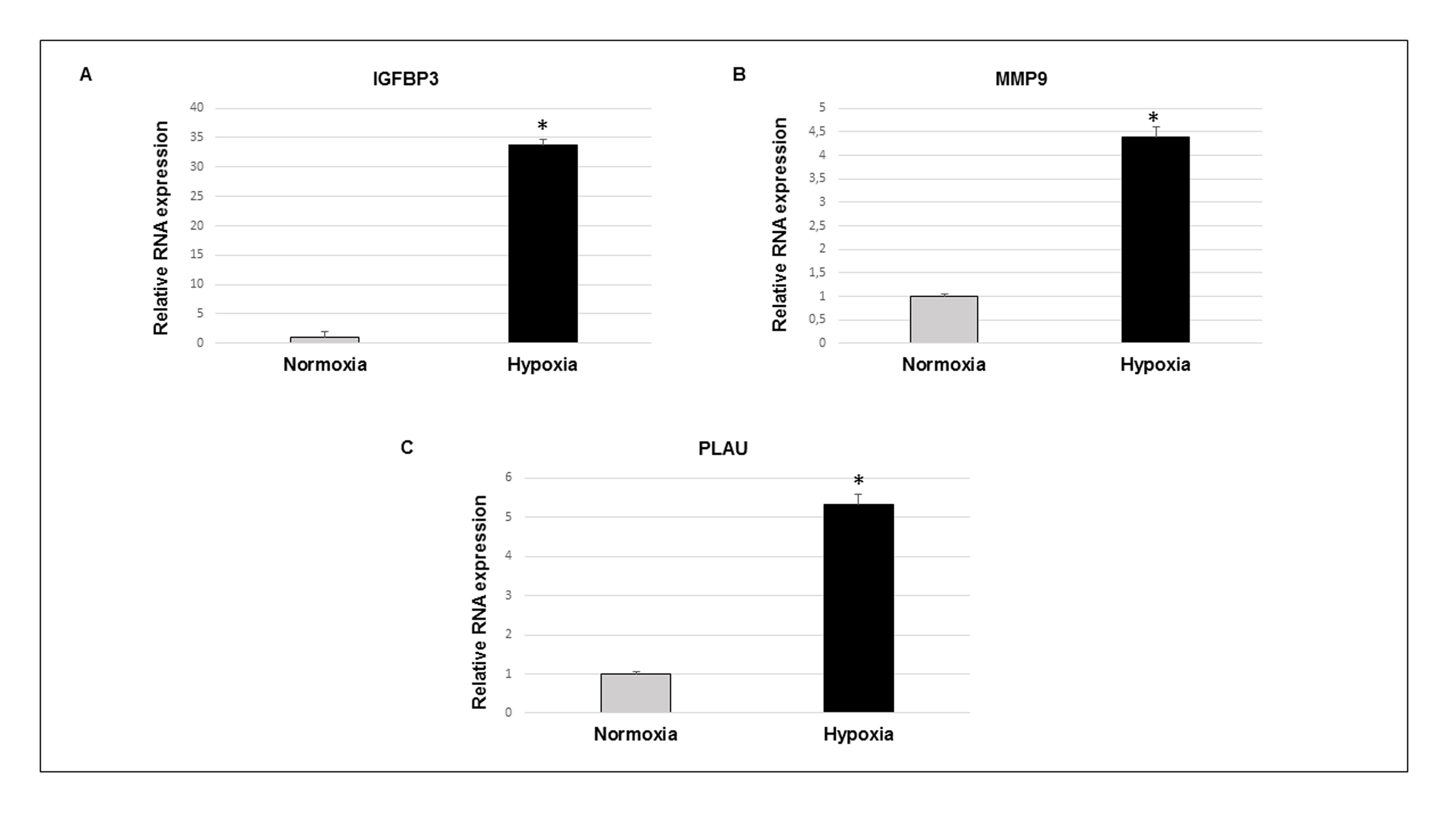

Supplement: Supplemental material [file bio143461_supp.zip › Supplemental Figure 2.tif]

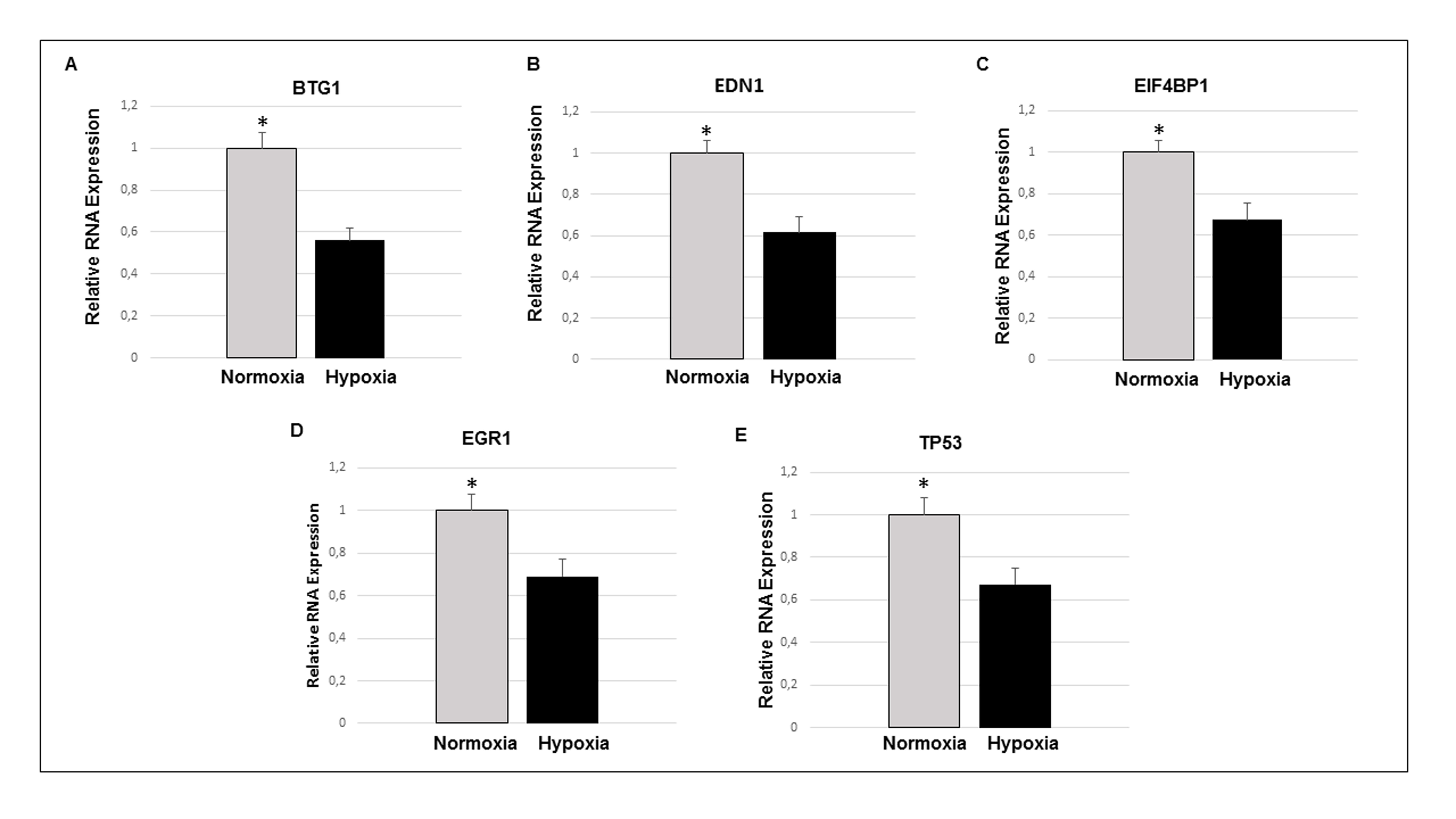

Supplement: Supplemental material [file bio143461_supp.zip › Supplemental Figure 3.tif]
